# The ice-breaker effect: singing mediates fast social bonding

**DOI:** 10.1098/rsos.150221

**Published:** 2015-10-28

**Authors:** Eiluned Pearce, Jacques Launay, Robin I. M. Dunbar

**Affiliations:** Social & Evolutionary Neuroscience Research Group, Department of Experimental Psychology, University of Oxford, Oxford, UK

**Keywords:** social cohesion, affect, endorphin, adult education, music

## Abstract

It has been proposed that singing evolved to facilitate social cohesion. However, it remains unclear whether bonding arises out of properties intrinsic to singing or whether any social engagement can have a similar effect. Furthermore, previous research has used one-off singing sessions without exploring the emergence of social bonding over time. In this semi-naturalistic study, we followed newly formed singing and non-singing (crafts or creative writing) adult education classes over seven months. Participants rated their closeness to their group and their affect, and were given a proxy measure of endorphin release, before and after their class, at three timepoints (months 1, 3 and 7). We show that although singers and non-singers felt equally connected by timepoint 3, singers experienced much faster bonding: singers demonstrated a significantly greater increase in closeness at timepoint 1, but the more gradual increase shown by non-singers caught up over time. This represents the first evidence for an ‘ice-breaker effect’ of singing in promoting fast cohesion between unfamiliar individuals, which bypasses the need for personal knowledge of group members gained through prolonged interaction. We argue that singing may have evolved to quickly bond large human groups of relative strangers, potentially through encouraging willingness to coordinate by enhancing positive affect.

## 1. Introduction

Creating and maintaining positive social relationships is essential for human physical and mental health and well-being (e.g. [1–4]). Furthermore, social support enhances the survival of an individual’s children, meaning that being part of a supportive social network may increase reproductive success [5,6]. Social networks provide practical and emotional support as well as providing a means of gaining new information and disseminating the cultural knowledge crucial for human survival [7–11]. Nonetheless, group living brings costs as well as advantages, for instance, competition for resources and an elevated risk of cuckoldry (e.g. [12]). For our ancestors, individuals who did not belong to a cohesive social group would have been unlikely to survive and, consequently, there is likely to have been strong selection for behaviours that create cohesive social units that remain together despite these costs [13,14].

Creating and maintaining personal relationships requires sufficient time investment, but finite time budgets place a limit on the number of personal relationships that an individual can maintain through one-on-one interactions [15–17]. Generating cohesion in large groups, therefore, requires some means of emotionally connecting many individuals simultaneously, without the need for direct dyadic interaction [18]. Such cohesion mechanisms are likely to be particularly important in modern humans, who are capable of sustaining much larger bonded social groups than any other primate [19,20]. However, the behaviours that have the capacity to perform this role are as yet not well understood. In this paper, we explore the role of group singing as one of these potential bonding behaviours, asking whether there is something special about singing, or whether any activity performed in a group context can have a similar bonding effect through providing opportunities for social engagement.

Singing is found in all human societies and can be performed to some extent by the vast majority of humans: singing is a universal human behavioural capacity, and this implies that it could have arisen as an evolutionary adaptation [21,22]. Notably, it has been argued that singing, as well as other musical activities, may have evolved as a mechanism of social bonding [23–25]. Support for this comes from the association between singing and the release of neuropeptides known to be associated with social bonding: oxytocin and β-endorphin [26–28]. β-Endorphin is implicated in mother–infant bonds, romantic relationships and social touch in humans [29–31], and appears to be released during synchronous behaviours that involve some physical exertion, such as rowing [32,33], laughter [34] and dancing [28], particularly in social contexts [35]. As a coordinated and often synchronous activity, for example, in terms of breath and heart rhythms, as well as timing and pitch [36], it is unsurprising that singing has also been linked with elevated β-endorphin levels [28].

In addition to the apparent endorphin effect, an expanding body of the literature has consistently shown that synchronous activity increases subsequent prosocial behaviour and feelings of affiliation (e.g. [37–45]). Furthermore, synchrony is interpreted by observers as a marker of high group cohesion and entitativity, suggesting that the association between synchrony and group unity is particularly strong [46,47]. Indeed, qualitative data from singing groups and choirs suggests that social interaction and a sense of belonging are important positive features of attendance [48–50]. Moreover, singing has been shown to increase positive affect and choir members often report a ‘lift’ in mood after singing [27,28,51–53]. This shared experience of positive mood enhancement can be seen as a further form of synchronization, preparing performers for further coordinated activity [54].

Overall, the universal nature of human singing and its consistent association with social behaviour suggests that it could have evolved as a mechanism of bonding social groups. However, as yet it is unclear whether there is something specific about singing that has a social bonding effect, or whether any activity that provides the opportunity for social engagement, particularly those that encourage laughter and thus β-endorphin release [34], would similarly lead to greater feelings of closeness towards a group. Furthermore, how the bonding process unfolds over time in singing versus other activities remains unexplored.

In collaboration with an adult education charity, the Workers’ Education Association (WEA), we followed up the participants attending either newly formed weekly singing classes or newly formed weekly non-singing classes (crafts or creative writing) over the course of seven months. We collected data at three timepoints (month 1, month 3 and month 7) and at each timepoint we asked participants to rate how close they felt to their class as a whole before and after they had taken part in the course activity (singing or crafts/writing). This allowed us to test the hypotheses that, compared with non-singers, singers would feel significantly closer to their group both after class compared with before class, and at the end of the seven-month period. In addition, we tested whether singers felt a greater lift in positive affect and a greater reduction in negative affect compared with non-singers after class compared with beforehand. Finally, we tested whether singers showed a greater increase in pain threshold (an indirect measure of endorphin release [28,32–34]) after class compared with beforehand, relative to non-singers.

## 2. Material and methods

### 2.1 Sample

The participants in this study comprised learners attending one of seven adult education classes (four singing classes, two creative crafts classes and one creative writing class) set up by the WEA specifically for the study. Participants ranged in age from 18 to 83 years (singers: *M*=60±12 years; non-singers: *M*=52±15 years). For the singing classes, 73 of 84 participants (87%) were female and for non-singing classes 45 of 51 participants (88%) were female. Across the three timepoints, the sample size decreased in both conditions (table 1) due to dropout. Twenty-seven participants (53%) in the non-singing condition and 48 participants (57%) in the singing condition provided data at all three timepoints. However, the statistical analysis used is not adversely affected by missing data, so the results presented here include the full sample.

**Table 1.** Descriptive statistics showing mean closeness (Inclusion of Other in Self score) before and after class, and the change between the two, at each timepoint for the two conditions, showing standard deviations in parentheses. The multi-level linear model (MLM) comparisons between the before and after scores for each condition are given for each timepoint.

			mean closeness to class (s.d. in parentheses)			MLM comparison between before and after scores		
condition	timepoint	*N*	before	after	change	*t*	d.f.	*p*-value
singing	1	64	2.53 (1.34)	4.33 (1.61)	1.80 (1.36)	10.47	58	<0.0001
	2	66	4.95 (1.40)	5.67 (1.27)	0.71 (0.91)	5.78	60	<0.0001
	3	58	5.50 (1.38)	6.07 (1.11)	0.57 (0.88)	4.31	53	<0.0001
non-singing	1	46	3.15 (1.83)	3.72 (1.70)	0.57 (1.05)	3.80	42	0.0005
	2	36	4.42 (1.54)	4.94 (1.59)	0.53 (0.97)	3.22	34	0.003
	3	32	5.53 (1.44)	5.78 (1.39)	0.25 (0.51)	2.74	28	0.011

### 2.2 Methods

There were two conditions in this study: singing and non-singing. The singing condition comprised four singing classes, who were taught by professional singing tutors using a Natural Voice Network (http://www.naturalvoice.net/) style approach. The non-singing comparison condition comprised two crafts classes and a creative writing class, who were also led by professional tutors. The tutors had been teaching their specialist subject for 2–20 years. Each class was 2 h long.

All seven classes were set up specifically for the study, so that although some participants knew each other from their local community, the class groups were newly formed at the start of the study. Data collection started during the second or third class of the course (i.e. the group had met once or twice prior to data collection at timepoint 1). The design of the study is that of a conventional quasi-experiment (field experiment) in which there is no control over the assignment of participants to experimental conditions. The classes ran over seven months comprising two terms with a two-week break in the middle. Data were collected at three timepoints: month 1 (timepoint 1), month 3 (immediately prior to the break; timepoint 2) and month 7 (timepoint 3).

At each of these three timepoints, participants completed a questionnaire before and after their class and provided a ‘pressure cuff measure’ (see below) before and after the class. At timepoint 1, participants were asked to provide demographic information as well as being asked whether they already knew anyone else in their class. In the non-singing condition, 14 participants (27%) knew no one before starting the class (known others *M*=2 others, range = 0–5 other people) and in the singing condition 24 participants (29%) reported knowing no one else before starting the class (known others *M*=2 others, range = 0–8 other people).

At each of the three timepoints, participants were asked to rate how close they felt to their class as a whole both before and after the class using a modified version of the pictorial Inclusion of Other in Self (IOS) 7-point scale from 1 to 7 [55], termed ‘closeness’ here. In addition, participants were asked to fill in the Positive and Negative Affect Schedule (PANAS) [56] both before and after the class. There were no significant differences in baseline (i.e. before the first class) closeness or positive or negative affect between singing and non-singing classes. Change in closeness/affect was calculated by subtracting the before-class score from the after-class score.

As direct measurement of β-endorphin release requires procedures that are impractical (PET scanning or lumbar puncture), it is standard practice to use a proxy measure [28,32–34]. Given the known function of β-endorphin as an analgesic, pain tolerance is often used: the greater the increase in pain threshold that an individual can withstand, the greater the implied level of circulating endorphins. Here we followed the procedure detailed in Dunbar *et al.* [34]: a sphygmomanometer (blood-pressure cuff) was gently inflated at a steady rate (10 mmHg s^−1^) until the participant first indicated that it felt ‘very uncomfortable’ (or a maximum pressure of 300 mmHg was reached). This measure was referred to as a ‘blood pressure cuff measure’ to avoid biasing participants. The same protocol was administered before and after the class at each of the three timepoints. An increase in pain threshold across an activity indicates endorphin activation. There was no significant difference in baseline (i.e. before the first class) pain thresholds between singing and non-singing classes. Participants who reached the maximum threshold (300 mmHg) at baseline, or were diabetic, had smoked or drunk alcohol in the last 12 h or had exercised in the last 2 h were excluded from the pain threshold analysis.

### 2.3 Analysis

Given that participants were nested in classes and therefore do not represent independent data-points, multi-level linear models (MLMs) were conducted for all analyses. These models control for extraneous variables such as the effect of the different tutors teaching the different classes. For within-subject comparisons of measures before and after a class, ‘individual participant’ was added as an additional nested layer. For initial comparisons between conditions, timepoint was included as a third nested layer because (i) for these analyses, we were interested in the difference between conditions rather than change over time and (ii) this maximized sample size while taking lack of independence into account. Note that for these analyses sample sizes do not correspond to numbers of participants, but the number of data-points included in each model, pooled across timepoints. For follow-up analyses looking at social bonding (IOS scores) over time, timepoint was included in the models as an independent factor.

## 3. Results

**Figure 1.**
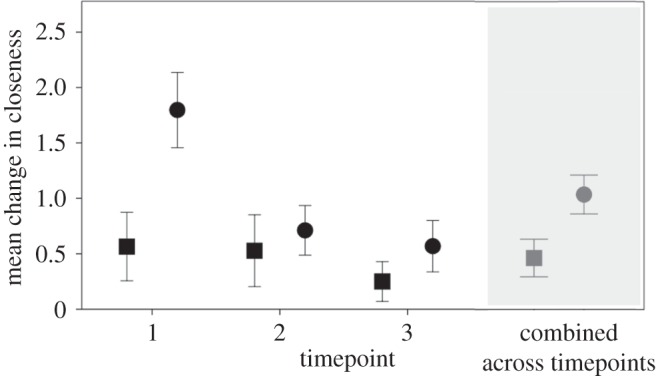
Mean change in closeness for singers (circles) and non-singers (squares), both separately across the three timepoints and pooled across the three timepoints (shaded grey box). Means are shown ±2 s.e.

Participants felt significantly closer to their classmates after class (singing: *M*=5.23, s.d.=1.64, *N*=197; non-singing: *M*=4.68, s.d.=1.79, *N*=114) compared with beforehand (singing: *M*=4.28, s.d. =1.89, *N* = 189; non-singing: *M*=4.23, s.d. =1.90, *N*=116): *t*_444.9_=3.486, *p*=0.0005. There was no significant main effect of condition (*p*=0.869), but there was a significant interaction between condition and the before/after comparison (*t*_447.7_=2.57, *p*=0.010). This interaction corresponds to a significantly greater increase in the change in closeness during a class (*t*_298.12_=5.01, *p*<0.0001) in the singing condition (*M*=1.04, s.d. =1.02, *N*=188) compared with the non-singing condition (*M*=0.46, s.d. =0.90, *N*=114). Singers thus experience a greater increase in closeness to their classmates than non-singers overall (figure 1). Nonetheless, both singers and non-singers demonstrated significant increases in closeness to their respective group at each of the three timepoints (table 1).

**Figure 2.**
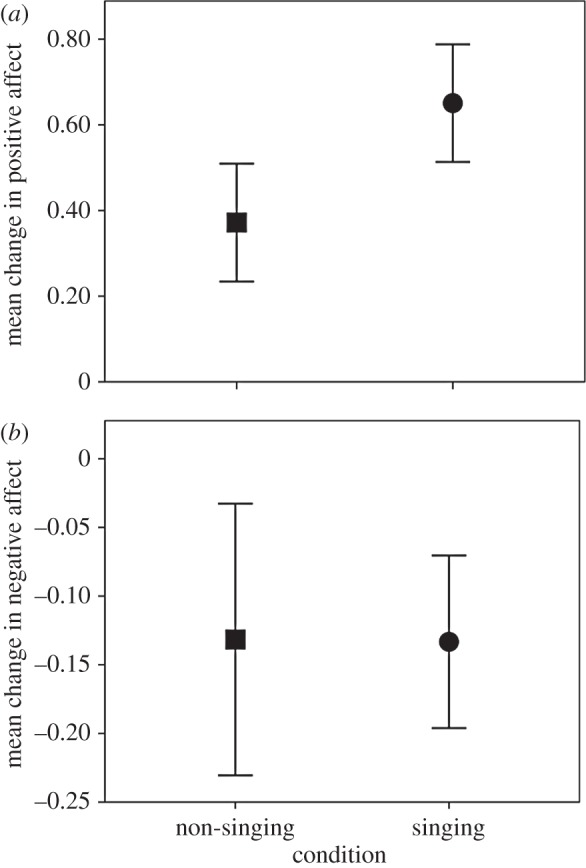
Mean change in (*a*) positive and (*b*) negative affect for singers (circles) and non-singers (squares), pooled across timepoints. Means are shown ±2 s.e.

Mean positive affect was significantly higher after class (singing: *M*=3.58, s.d. =1.04, *N*=197; non-singing: *M*=3.50, s.d.=0.99, *N*=113) compared with beforehand (singing: *M*=2.93, s.d.=0.97, *N*=189; non-singing: *M*=3.13, s.d.=0.92, *N*=116): *t*_479.1_=4.19, *p*<0.0001. There was no significant main effect of condition (*p*=0.249). However, the interaction between condition and the before/after contrast was significant: *t*_480.1_=2.79, *p*=0.005. This interaction corresponds to a significantly greater increase in the change in positive affect during a class (*t*_298_=2.70, *p*=0.007) in the singing condition (*M*=0.65, s.d.=0.94, *N*=187) compared with the non-singing condition (*M*=0.37, s.d.=0.73, *N*=113) (figure 2*a*).

Negative affect was significantly lower after class (singing: *M*=1.11, s.d.=0.29, *N*=193; non-singing: *M*=1.21, s.d.=0.51, *N*=111) compared with beforehand (singing: *M*=1.24, s.d.=0.51, *N*=188; non-singing: *M*=1.34, s.d.=0.76, *N*=115): *t*_453.4_=−2.47, *p*=0.014. There was no significant main effect of condition (*p*=0.071) and no significant interaction between condition and the before/after comparisons (*p*=0.824). Correspondingly, change in negative affect did not differ significantly between the conditions (*p*=0.888) (figure 2*b*).

**Figure 3.**
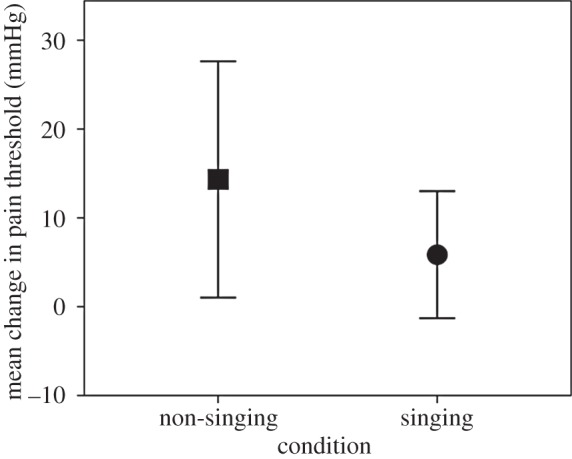
Mean change in pain thresholds for singers (circles) and non-singers (squares), pooled across timepoints. Means are shown ±2 s.e.

Pain thresholds were significantly higher after class (singers *M*=182.75, s.d.=63.04, *N*=103; non-singers *M*=161.33, s.d.=61.12, *N*=45) compared with beforehand (singers *M*=176.89, s.d.=58.05, *N*=103; non-singers *M*=147.00, s.d.=60.88, *N*=45): *t*_210.18_=2.22, *p*=0.027. There was no main effect of condition (*p*=0.427) and no significant interaction between condition and the before/after comparisons (*p*=0.274). Correspondingly, change in pain threshold did not differ significantly between the conditions (*p*=0.474) (figure 3).

At the end of the classes (timepoint 3), no significant difference in social closeness scores was found between singers and non-singers after class: *p*=0.748, (table 1). However, there was a main effect of singing on change in closeness (*t*_296_=6.28, *p*<0.0001) and an interaction between singing and timepoint (interaction between condition and (i) contrast between timepoint 1 and timepoint 2: *t*_296_=−3.65, *p*=0.0003; (ii) timepoint 1 and timepoint 3: *t*_296_=−3.07, *p*=0.002). To clarify these interaction effects, we tested models for each timepoint separately. These demonstrated that although the positive change in reported closeness was significantly greater for singers than non-singers at timepoint 1 (*t*_4.12_=4.32, *p*=0.012), there was no significant difference between the two conditions at timepoint 2 (*p*=0.503) or timepoint 3 (*p*=0.165) (figure 1).

**Figure 4.**
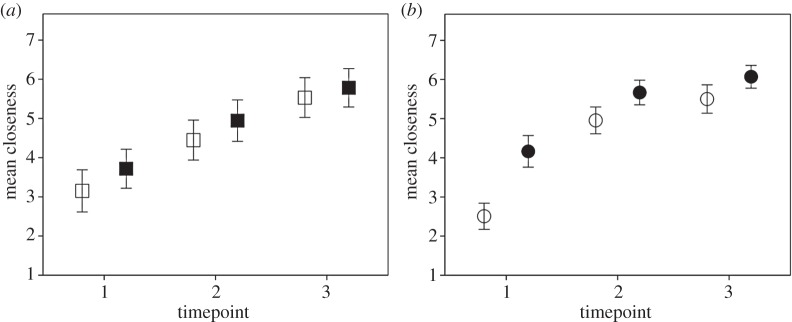
(*a*) Mean closeness scores before (open squares) and after (filled squares) class for non-singers across the three timepoints and (*b*) mean closeness scores before (open circles) and after (filled circles) class for singers across the three timepoints. Means are shown ±2 s.e.

Figure 4 suggests that whereas the pattern of increase in closeness towards classmates within and across timepoints increases linearly for non-singers, the positive relationship over time is more curved for singers: a sharper increase initially, which levels off. This is supported by the finding that although the change in closeness for singers was significantly greater at timepoint 1 than timepoint 2 (*t*_182.9_=5.75, *p*<0.0001), there was no difference in the change in closeness between timepoints 2 and 3 (*p*=0.460) (figure 1 and the mean changes given in table 1). For non-singers, on the other hand, the change in closeness was not different between either timepoints 1 and 2 (*p*=0.852) or timepoints 2 and 3 (*p*=0.208) (figure 1 and the mean changes given in table 1): for non-singers the change over the course of a class was consistent over time.

We fitted linear and quadratic curves to the data in the two conditions separately, taking the nested class structure into account, and tested for a difference in model fit, measured as maximum likelihood. For both singers (*t*_381.40_=15.20, *p*<0.0001) and non-singers (*t*_226.23_=10.01, *p*<0.0001), a significant positive linear relationship was found between time (coded 1–6 to take into account both the before and after measures and timepoints) and closeness score. By contrast, in a quadratic model only singers showed a significant partial relationship between the squared (quadratic) term and closeness (*t*_380.20_=−6.08, *p*<0.0001), whereas the non-singers did not (*p*=0.338), implying that only the linear term was relevant for non-singers (partial relationships for the linear term: non-singers: *t*_225.05_=3.03, *p*=0.003; singers *t*_380.20_=9.24, *p*<0.0001). Goodness-of-fit tests revealed that the quadratic model fitted the singing data significantly better than the linear model (*χ*^2^=35.42, *p*<0.0001), but that there was no difference in fit between the linear and quadratic models for the non-singing data (*p*=0.335).

## 4. Discussion

Overall our results indicate that compared with individuals participating in craft or creative writing classes, singers experience a greater increase in both self-reported closeness to their group and positive affect. By contrast, although negative affect decreased and pain thresholds (a proxy for endorphin release) increased during classes, there was no difference in these changes between the conditions: participants could withstand more pain and reported lower negative affect after class compared with beforehand, but, contra our hypotheses, this effect occurred irrespective of the activity that they had performed.

Despite the fact that singers and non-singers reached similar levels of closeness to their classmates by the end of the study (table 1), comparisons revealed distinctly different patterns in how group bonding occurred. Whereas singers showed a significantly greater boost in closeness at timepoint 1 compared with non-singers (reflected in the greater change found in the pooled data) followed by a plateau, non-singers showed a more gradual, linear increase to reach the same point eventually. Contrary to our hypothesis that singing would lead to closer groups overall, therefore, the distinguishing feature of singing was that it bonded groups more quickly than the other activities.

The differences in the temporal patterns of bonding suggest that the singing and non-singing classes may have reached similar levels of emotional closeness through different mechanisms, or at least different combinations of mechanisms. Building on a growing body of the literature, we propose that group unity depends on behaviours that are synchronous and involve some muscular effort, which trigger the release of neuropeptides such as β-endorphin, yield enhanced positive affect, and in turn may enhance individuals’ willingness to cooperate [28,32,34,35,57]. By contrast, building close personal ties with individuals relies more on frequently repeated one-on-one (or small group) interactions in which individuals have the opportunity to talk and observe each other at close quarters to build up an idea of a potential social partner’s trustworthiness and usefulness as a coalition partner [15]. We argue that, in the singing classes, shared musical activity initially facilitated group bonding by bypassing the need to get to know everyone in the class individually, creating general feelings of positivity towards everyone present. Further closeness in the singing classes may have arisen as new relationships were built with individual classmates during the tea-break conversations or between classes.

On the other hand, the non-singing classes provided more opportunity than the singing classes for talking to each other, but lacked a powerful means of bonding a whole class simultaneously. This may account for the more steady increase in group closeness over time in the comparison condition classes, reflecting the gradual strengthening of personal ties with individual group members associated with regular and repeated interactions. Interpersonal closeness may have been amplified through laughter, which has been shown to lead to greater pain threshold increase (endorphin release) in social versus solitary settings [34], but is limited to bonding no more than three individuals at a time in typical conversational interactions [58]. In the non-singing classes, therefore, group connectedness is likely to have culminated from the building of personal relationships through conversational interaction and associated laughter, whereas singing may have led to the development of personal relationships within the context of an already heightened sense of group cohesion. As singers reached the ceiling of the IOS measure more quickly than non-singers, it is possible that in reality singers did not reach a plateau in emotional closeness to their classmates, but that this continued to deepen over time beyond the level experienced by non-singers. Additional social bonding measures would be required to test this possibility.

It should be noted that in contrast with the singing classes, learners in the craft and creative writing classes worked on individual projects rather than working together towards a shared goal. This means that this study did not distinguish between the group-bonding effects of the physical act of singing *per se* and the existence of a shared group motivation to create a piece of music together. Further work should test between these explanations by comparing singing classes with, for instance, groups cooperating to produce a piece of drama or a joint craft project such as a collaborative quilting.

Although protracted interaction is likely to be necessary in order for intimate personal relationships to develop within a group, singing may be able to kick start this process in humans: singing breaks the ice so that individuals feel closer to the group as a whole even if they do not yet know anything about the individual members. Such an effect may overcome time constraints on the creation of individual relationships to allow large human groups to coordinate effectively and quickly. In this regard, it is interesting that religion, another potential mechanism for connecting large numbers of individuals, often incorporates singing or chanting in groups [18]. The capacity of singing to bond groups of relative strangers in humans may have played a crucial role in allowing modern humans to create and maintain much larger social networks than their evolutionary relatives, which in turn may have facilitated the colonization of risky environments across the globe [59].

## Supplementary Material

PEARCE_et_al_ESM.xlsx - Data file

## Data accessibility

The data supporting this article have been uploaded as part of the electronic supplementary material.
